# The First Human Epitope Map of the Alphaviral E1 and E2 Proteins Reveals a New E2 Epitope with Significant Virus Neutralizing Activity

**DOI:** 10.1371/journal.pntd.0000739

**Published:** 2010-07-13

**Authors:** Ann R. Hunt, Shana Frederickson, Toshiaki Maruyama, John T. Roehrig, Carol D. Blair

**Affiliations:** 1 Microbiology, Immunology and Pathology Department, Colorado State University, Fort Collins, Colorado, United States of America; 2 Alexion Antibody Technologies, San Diego, California, United States of America; 3 Division of Vector-Borne Infectious Diseases, Centers for Disease Control, Fort Collins, Colorado, United States of America; University of Texas Medical Branch, United States of America

## Abstract

**Background:**

Venezuelan equine encephalitis virus (VEEV) is responsible for VEE epidemics that occur in South and Central America and the U.S. The VEEV envelope contains two glycoproteins E1 (mediates cell membrane fusion) and E2 (binds receptor and elicits virus neutralizing antibodies). Previously we constructed E1 and E2 epitope maps using murine monoclonal antibodies (mMAbs). Six E2 epitopes (E2^c,d,e,f,g,h^) bound VEEV-neutralizing antibody and mapped to amino acids (aa) 182–207. Nothing is known about the human antibody repertoire to VEEV or epitopes that engage human virus-neutralizing antibodies. There is no specific treatment for VEE; however virus-neutralizing mMAbs are potent protective and therapeutic agents for mice challenged with VEEV by either peripheral or aerosol routes. Therefore, fully human MAbs (hMAbs) with virus-neutralizing activity should be useful for prevention or clinical treatment of human VEE.

**Methods:**

We used phage-display to isolate VEEV-specific hFabs from human bone marrow donors. These hFabs were characterized by sequencing, specificity testing, VEEV subtype cross-reactivity using indirect ELISA, and *in vitro* virus neutralization capacity. One E2-specific neutralizing hFAb, F5n, was converted into IgG, and its binding site was identified using competitive ELISA with mMAbs and by preparing and sequencing antibody neutralization-escape variants.

**Findings:**

Using 11 VEEV-reactive hFabs we constructed the first human epitope map for the alphaviral surface proteins E1 and E2. We identified an important neutralization-associated epitope unique to the human immune response, E2 aa115–119. Using a 9 Å resolution cryo-electron microscopy map of the Sindbis virus E2 protein, we showed the probable surface location of this human VEEV epitope.

**Conclusions:**

The VEEV-neutralizing capacity of the hMAb F5 nIgG is similar to that exhibited by the humanized mMAb Hy4 IgG. The Hy4 IgG has been shown to limit VEEV infection in mice both prophylactically and therapeutically. Administration of a cocktail of F5n and Hy4 IgGs, which bind to different E2 epitopes, could provide enhanced prophylaxis or immunotherapy for VEEV, while reducing the possibility of generating possibly harmful virus neutralization-escape variants *in vivo*.

## Author Summary

Although the murine immune response to Venezuelan equine encephalitis virus (VEEV) is well-characterized, little is known about the human antibody response to VEEV. In this study we used phage display technology to isolate a panel of 11 VEEV-specfic Fabs from two human donors. Seven E2-specific and four E1-specific Fabs were identified and mapped to five E2 epitopes and three E1 epitopes. Two neutralizing Fabs were isolated, E2-specific F5 and E1-specific L1A7, although the neutralizing capacity of L1A7 was 300-fold lower than F5. F5 Fab was expressed as a complete IgG1 molecule, F5 native (n) IgG. Neutralization-escape VEEV variants for F5 nIgG were isolated and their structural genes were sequenced to determine the theoretical binding site of F5. Based on this sequence analysis as well as the ability of F5 to neutralize four neutralization-escape variants of anti-VEEV murine monoclonal antibodies (mapped to E2 amino acids 182–207), a unique neutralization domain on E2 was identified and mapped to E2 amino acids 115–119.

## Introduction

Venezuelan equine encephalitis virus (VEEV) is a member of the *Alphavirus* genus in the family *Togaviridae* and is maintained in a natural enzootic cycle between mosquitoes and rodent hosts, although equines and humans may also be infected in an epizootic cycle [1]. While VEEV causes high-titered viremia in equines, resulting in encephalitis with a mortality rate between 30 to 90%, disease in humans is usually self-limited and consists of fever, chills, malaise, and severe headaches, with 1 to 4% progressing to severe encephalitis [2]. Outbreaks of epizootic VEEV (subtypes 1AB and 1C) occur periodically in South and Central America, even spreading to south Texas, and therefore it is considered an emerging pathogen [3]. While the last major transcontinental outbreak of epizootic VEEV occurred in 1969–1971, smaller South and Central American outbreaks of VEEV 1C have occurred since, such as the one in Colombia and Venezuela in 1995–1996 [4], [5]. In the mid to late 1990's outbreaks caused by the usually enzootic subtype VEEVs occurred in Peru and Panama (VEEV-1D), and in the Mexican states of Chiapas and Oaxaca (VEEV-1E) [6]–[9].

VEEV has potential as a bioweapon, principally because of its low human infective dose, easy production, and capability for effective transmission by aerosolization [10], [11], and it is listed as a NIAID Category B priority pathogen. Experimental vaccines (TC-83, C-84) have been used to protect laboratory personnel and military troops, but are not licensed for general use [12]–[14]. A new, live-attenuated vaccine, V3526, developed from a virulent VEEV infectious clone by site-directed mutagenesis [15], proved effective in animal studies [16]–[18], but was associated with adverse events in phase 1 clinical trials and subsequently abandoned [19], [20].

Alphaviruses have a positive-sense, single-stranded RNA genome of approximately 11.45 kb enclosed within an icosahedral nucleocapsid surrounded by a lipid bilayer derived from the infected cell's plasma membrane. Two integral membrane glycoproteins, E1 and E2, are embedded in the lipid envelope and are assembled as heterodimers into 80 trimeric spikes on the virus surface [21]–[25]. Although the crystal structures of the E1 and capsid proteins of several alphaviruses have been solved, no well-diffracting crystals of either E2 or virus particles have been obtained [26]–[28]. However, cryo-electron microscopy (cryoEM) reconstructions of several alphaviruses have been reported and have provided insights into probable E1/E2 structure-function relationships [25], [29]–[32]. The E1 glycoprotein is responsible for cell membrane fusion, while E2 is primarily involved in receptor binding and cell entry as well as eliciting VEEV-specific neutralizing antibodies.

We have previously analyzed the antigenic structure of both the VEEV E1 and E2 glycoproteins using murine (m) monoclonal antibodies (MAbs) and defined six E2 epitopes (E2^c,d,e,f,g,h^) involved in VEEV neutralization [33]–[35]. These epitopes clustered in a “critical VEEV E2 neutralization site,” and were mapped to E2 amino acids (aa) 182–207 by sequencing the RNA of MAb neutralization-escape VEEV variants [36]. Similar E2 neutralization sites have been identified for both Sindbis virus (SV) (E2 aa170–220) and Ross River virus (RRV) (E2 aa216–251) using mMAbs [37]–[39]. The VEEV epitopes E2^c^ and E2^h^ are the most conserved on the E2 glycoproteins of heterologous VEEVs [33], [40].

Specific treatment for VEEV infections is not available; however, MAbs reacting with the critical neutralization site demonstrate potent protective activity in a murine model following either peripheral or aerosol challenge with virulent VEEV [12], [35], [41], [42]. Moreover, anti-E2^c^ mMAb 1A4A-1 and anti-E2^g^ mMAb 1A3A-9, as well as the humanized anti-E2^c^ mMAb Hy4 IgG, have been shown to provide post-exposure protection when administered within 24 hr after virus inoculation [12], [43].

Fully human (h) MAbs would be the best choice for clinical treatment of human infections; however, little is known about the immunologic specificities of the human antibody repertoire to VEEV, and no protective hMAbs have yet been isolated, characterized, or implemented. In this report we have presented a map of the human VEEV antibody response and determined the human immunodominant epitopes on the VEEV E1 and E2 proteins. We used phage-display technology to isolate VEEV-specific hFabs from bone marrow donors known to have circulating antibodies for VEEV [44]–[47]. We have characterized a panel of 11 hFabs for VEEV surface protein specificity, subtype cross-reactivity, and *in vitro* virus-neutralizing capacity. Two anti-E2 hFabs, H6 and F5, one of which (F5) exhibited potent neutralizing activity, were converted to fully human IgG1 molecules. Two F5 hMAb neutralization-escape VEEV variants were isolated. Sequencing of the E1 and E2 protein genes of these variant viruses identified a unique human VEEV neutralization domain as the likely binding site of the F5 hMAb. Alphavirus cryoEM maps, with associated markers, support proposing a probable surface-accessible location on VEEV E2 for the F5 native (n) IgG binding site.

## Methods

### Viruses and murine MAbs

The VEE complex viruses used in this study were Trinidad donkey (TrD, subtype 1, variety AB), vaccine strain TC-83 (1-AB), P676 (1-C), 3880 (1-D), Mena II (1-E), Everglades (EVE)(2), Mucambo (MUC) (3-A), Pixuna (PIX) (4), Cabassou (CAB) (5), and AG80-663 (Rio Negro) (6), which were obtained from the Division of Vector-Borne Infectious Diseases (DVBID), Centers for Disease Control (CDC), Fort Collins, CO. Viruses grown in Vero cells were purified by equilibrium density-gradient centrifugation [48]. Purified VEEV TC-83 used for panning phage display libraries was inactivated with 0.05% or 0.3% β-propiolactone (BPL) in 0.1M Tris base, pH 9, for 48 h at 4°C. Virus inactivation was verified by inoculation of Vero cells, which were monitored for cytopathic effects (CPE). Inactivated virus was evaluated by ELISA for preservation of important epitopes reactive with neutralizing mMAbs [33]. Anti-VEEV neutralizing mMAbs (3B4C-4, 1A4D-1, 1A3A-9, 1A3B-7, and 3B2A-9) and their respective neutralization-escape variant viruses used in the characterization of hMAb F5 nIgG have been well-documented [33], [34], [36].

### Phage library creation, Fab selection and production of human anti-VEEV Fabs and MAbs

Total RNA was obtained from bone marrow and blood samples supplied by two military donors (951, 1037) using Tri-reagent BD (Molecular Research Center, Inc., Cincinnati, OH) according to the manufacturer's instructions. Blood utilized in this project was obtained under protocol 01-124 approved by Scripps Clinic, IRB#00001283, Assurance Number FWA00000467. Bone marrow samples were obtained from a commercial source. Donor sera ELISA titers to VEEV TC-83 were 1∶500–3000.

Messenger RNA was isolated using Oligotex spin columns (Qiagen, Valencia, CA) following the manufacturer's protocol. To amplify mRNA, first strand cDNA was synthesized using SuperScript II reverse transcriptase (Invitrogen, Carlsbad, CA) according to the manufacturer's protocol. Second strand cDNA was synthesized according to the method fully described in the patent WO2005/060641A2 [49]. The cDNA was purified with a PCR purification kit (Qiagen) and single primer amplification was performed according to the method fully described in the patent. The amplified products were digested with Xba I/Sac I for the kappa light chains (LCs), Xba I/Kas I for the lambda LCs, and Xho I/Age I (Pin AI) for the heavy chains (HCs), and cloned into Fab expression phagemid vectors PAX243hGK and PAX243hGL. Two Fab libraries were generated for each donor, one expressing kappa LCs and one, lambda LCs, and both utilizing gamma HCs.

Fab-bearing phage from all libraries were panned through one to four rounds of enrichment against inactivated VEEV TC-83 coated in 96-well plates overnight at 4°C. Wells were washed with water and blocked 1 h at 37°C with 3% bovine serum albumin (BSA) in phosphate-buffered saline (PBS). The phage library was added to the wells and incubated at 37°C for 2 h. Wells were washed 10 times with PBS containing 0.05% Tween-20 (PBS-T). The bound Fab-phage were eluted with 2M glycine, pH 2.2, neutralized with 2M Tris base, used to infect log phase *E. coli* cells, strain ER 2738, and amplified by adding helper phage, strain VCSM13, to the infected bacteria for each round of panning. Individual colonies were produced by plating infected bacteria.

Screening by ELISA on inactivated VEEV TC-83 virus was performed in high throughput mode using a Tecan robot (Tecan Systems Inc., San Jose, CA) for large numbers of colonies (>1000) picked using a Q-pix instrument (Genetix Inc., Boston, MA). Individual colonies were grown overnight in deep-well microtiter dishes in a Hi-Gro high-speed incubator-shaker (GeneMachines, San Carlos, CA). Aliquots were removed and stored as stocks containing 15% glycerol or 10% DMSO. After centrifugation of the deep-well dishes, Fab-containing supernatants were collected for ELISA screening. Alkaline phosphatase (AP)-labeled goat anti-human Fab (Pierce Protein-Thermo Fisher Scientific, Rockford, IL) was used to detect expressed Fab bound to antigen. Miniprep DNA (Qiagen) from positive colonies was sequenced across the light and heavy chains using standard primers for the phagemid expression vectors. Sequences were analyzed using DNAstar software (DNAstar Inc., Madison, WI) to identify and classify unique candidates. For soluble Fab expression and purification, the gene III fusion region of the phagemid was removed from positive, unique candidates by subcloning. At this stage it was possible to insert an oligonucleotide that encoded a combination epitope tag consisting of an influenza virus hemagglutinin (HA tag) and 6 histidine residues (His tag) for detection and purification with anti-HA and/or Ni-NTA [50].

Panning and screening of additional libraries were done on inactivated VEEV TC-83, either with or without epitope masking by non-neutralizing hMAbs F2 engineered (e) IgG and H6 eIgG to increase the chances of isolating Fabs with neutralizing capability. For panning with epitope masking, wells coated with VEEV TC-83 were incubated with F2 eIgG (50 µg/ml) and H6 eIgG (10 µg/ml) in 1% BSA-PBS at 37°C for 30 min followed by addition of library phage and incubation at 37°C for 1.5 h.

### Fab/MAb expression and purification

Fabs selected for characterization were cloned into a Fab expression vector, PAEV1, using Eco RI/Spe I restriction sites available in the vector (Figure S1A). Two Fabs, K1B11 and K1H3, which had Eco RI sites in their LCs were cloned into a PAEV1 vector containing Fab L1A7 LCs and HCs by using the Xba I/Age I sites (Figure S1B). The selected Fabs were produced in BL21 *E. coli* cells with induction by isopropyl-β-D-thiogalactopyranoside and purified on a goat anti-human IgG F(ab′)_2_ affinity column. Purified F5 Fab for use as a positive control in ELISA was prepared from F5 eIgG by papain digestion using an ImmunoPure Fab Preparation Kit (Pierce) according to the manufacturer's instructions.

Four hFabs (F2, F5, G1 and H6) were converted to full-length IgG1s in a two-step cloning process (Figure S2). The region between LC and HC (Not I to Xho sites) was replaced with mammalian control elements, including a poly A signal for the end of the LC, a human cytomegalovirus promoter to direct expression of the HC, and an HC mammalian leader sequence. This intermediate was inserted via restriction sites Sfi I and Age I into a mammalian expression vector containing additional control elements and IgG1 HC constant domains. The resulting “engineered” IgG1 differed from the n sequence due to the few non-n aa codons at three restriction sites (Xba I, Sac I, and Xho I) present at the N-terminus of both LC and HC. F5 eIgG was then converted to IgG containing n sequences in three steps using site-specific mutation and overlap PCR (Figure S3). The sequences of all constructs were verified.

HMAbs were produced by transfecting 120 ml of 293-EBNA cells (3.75×10^5^ cells/ml) with 64 µg of MAb expression vector DNA using Effectene (Qiagen) according to the manufacturer's protocol. The culture supernatant was harvested 5–7 days after transfection and each MAb was purified on a protein A column using FPLC. Stable cell lines producing F5 nIgG were established by selecting transfected 293-EBNA cells with puromycin (10µg/ml). The transfected cells were initially grown in 24-well plates and medium was screened by ELISA using a goat anti-human IgG F(ab′)_2_ antibody-AP conjugate (Jackson ImmunoResearch, West Grove, PA) for detection. The 2 wells with the highest antibody expression were chosen for single cell selection in four 96-well plates. Wells containing single-cell colonies were tested by ELISA and the best producers were chosen for expansion. One of these clones, IE9, was grown in 1.5 L and 6 L cultures that produced 25 mg and 50 mg MAb, respectively, after protein A column purification. The purified antibody showed specific binding to inactivated VEEV TC-83 that was comparable to that of F5 nIgG prepared from transient transfection of 293-EBNA cells.

The GenBank accession numbers for the F5 nIgG light and heavy chain variable region sequences are HM047070 and HM047071, respectively.

### Serological tests for Fabs and MAbs

Indirect ELISAs were used to verify VEEV binding by purified hFabs and to determine the cross-reactivity of selected hFabs and hMAbs to six VEEV subtypes and four varieties of subtype 1. ELISAs were performed essentially as previously described [43], [51]. Fab or MAb binding to purified virus was detected by goat anti-human IgG F(ab′)_2_- or Fc-specific-AP conjugates (Jackson ImmunoResearch). An absorbance ratio (A_405_ test sample/A_405_ negative control) >2 was considered to be positive.

Competitive binding assays (CBAs) were used to determine the ability of F5 nIgG to block the binding of anti-VEEV neutralizing mMAb-horseradish peroxidase (HRP) conjugates, and, conversely, the ability of purified mMAbs or hFabs to block the binding of F5 nIgG-HRP to virus. A dilution of each unconjugated mMAb, hMAb, or hFab that resulted in approximately 70% binding to VEEV TC-83 was determined by titration in an indirect ELISA, using either AP-conjugated goat anti-mouse IgG (Fc-specific) or goat anti-human IgG [F(ab′)_2_-specific] as a detector and Sigma 104 phosphatase substrate. Reactions were read at 405 nm. Dilutions of MAb conjugates that resulted in an A_405_ of approximately 1.0 were also determined for use in CBAs. For competitions using MAb-HRP conjugates, wells were coated with 1 µg VEEV TC-83 overnight and then blocked with 3% goat serum in PBS for 1 h at 37°C. MAb or Fab competitors were added at predetermined dilutions, serially diluted 1∶2 in PBS-T, and incubated at room temperature for 30 min. A previously determined constant dilution of MAb-HRP in blocking buffer was added to each serially-diluted competitor and incubated for 1 h at 37°C. One hundred µl premixed tetramethyl benzidine substrate (Neogen Corp., Lexington, KY) was added to each well, the reaction was stopped in 15–30 min with 50 µl 1N H_2_SO_4_, and plates were read at 450 nm.

Viral protein specificity of each antibody was determined by immunoblot using precast 8% Tris-glycine gels (Invitrogen) for fractionation of purified VEEV TC-83, 1 or 2 µg per lane, under both reducing and non-reducing conditions for 110 min at 125 volts. Electroblotting onto 0.2 µm nitrocellulose membranes using a Novex XCell blotting apparatus (Invitrogen) was done according to the manufacturer's protocol. Membranes were blocked with StartingBlock buffer (Pierce) at 4°C to prevent nonspecific binding. Membrane strips were incubated with the purified hMAbs and hFabs as well as protein E1- and E2-specific mMAbs for 2 h at room temperature, followed by incubation with either AP-conjugated goat anti-human IgG F(ab′)_2_ or goat anti-mouse IgG (Jackson ImmunoResearch) for 1 h, and then visualized with 5-bromo-4-chloro-3-indolyl-phosphate/nitroblue tetrazolium phosphatase substrate (Kirkegaard and Perry Laboratories, Gaithersburg, MD). The PRNT for IgG or Fab antibody fragments was performed essentially as previously described [43].

### Selection and genome sequencing of F5 nIgG neutralization-escape variants of VEEV TC-83

Neutralization-escape variant viruses were selected with F5 nIgG using an infectious clone of VEEV TC-83, pVE/IC-92, in a manner similar to that used previously to select escape variant viruses for neutralizing mMAbs [36], [52]. In this case, different amounts of purified MAb F5 nIgG (6 replicates each of 50 µg, 5 µg, and 0.5 µg) were incubated with approximately 100 pfu of virus for 1 h at 37°C and then plated on Vero cell monolayers in 6-well plates. Well-isolated plaques were cored, eluted overnight at 4°C, and 0.5 ml of the eluted virus from each sample was incubated with 25 µg (5 ml) of F5 nIgG for 1 h at 37°C. Each sample was then adsorbed to Vero cells containing 50 µg MAb/ml in the culture medium and monitored for CPE. Supernatant was collected from this second selection cycle from wells showing CPE and the virus seed was passed once in Vero cells without addition of F5 nIgG.

Two variant viruses were isolated and RNA was extracted from 140-µl aliquots of seed virus from the variant and parent viruses using the QIAamp viral RNA Kit (Qiagen) following the manufacturer's instructions. RNA was eluted in 30–40 µl of nuclease-free water and stored at −80°C. Amplimers for sequencing the glycoprotein-coding region of pVE/IC-92 and the two variant viruses were generated by standard RT-PCR performed with either 5 or 10 µl of template RNA and 20 pmol of each primer (Table S1) in a 50 µl-reaction using the Titan RT-PCR Kit (Roche Molecular Biochemicals, Indianapolis, IN), following the manufacturer's protocol. Initial RT was performed at 50°C for 30 min, followed by denaturation at 96°C for 2 min. PCR included 10 cycles of 96°C for 20 sec, 52°C for 30 sec, 68°C for 2.5 min; 25 cycles of 96°C for 20 sec, 52°C for 30 sec, 68°C for 2.5–6.7min; and a final extension at 68°C for 7 min in a DNA Engine Thermocycler (BioRad, Hercules, CA). The resulting amplimers were gel purified using a QIAquick Gel Extraction Kit (Qiagen), essentially following the manufacturer's protocol. Approximately 20–30 ng of each amplimer was used for direct sequencing using a 9800 Fast Thermocycler, a Big Dye automated DNA sequencing kit, and a 3130×l Genetic Analyzer (Applied Biosystems, Foster City, CA).

## Results

### Generation of human MAbs and Fabs for VEEV

Bone marrow specimens with matched sera donated by VEEV seropositive, active service military personnel were provided to Alexion Antibody Technologies (AAT), San Diego, CA, from a commercial source. The human donor sera were tested against inactivated VEEV TC-83 using an indirect ELISA. Only inactivated VEEV was used at AAT; both native and inactivated VEEV were used at DVBID, CDC, and Colorado State University, Fort Collins, CO. Two donor bone marrows, 951 and 1037, were selected for use in constructing phage display libraries. Large libraries, 2.6–5.7×10^9^ phage particles per ml, were obtained and panned through four rounds on inactivated VEEV TC-83. A panel of hFab clones from panning rounds three and four for each of four libraries (951κ, 951λ, 1037κ, and 1037λ) was screened on VEEV TC-83 as well as 1% BSA (negative control). Three hFab clones with the highest ELISA signals from each of the four libraries were selected for sequence analysis. The sequencing results (not shown) showed that all three 951κ clones were identical. Ten of the 12 clones had the same variable HC region, but the majority of those Fabs had different LC sequences. In all, there were 10 unique clones in three separate HC groupings. Four hFab clones, P3F2, P3F5, P3H6, and P3G1, were expressed as soluble molecules and purified by Ni-NTA column chromatography for use in serological assays. These four clones were also converted to full-length IgG1 molecules (Figure S2). Because the P3F5 clone had significant VEEV-neutralizing ability, a stable cell line expressing F5 nIgG was generated in 293-EBNA cells.

An attempt was made to identify additional anti-VEEV hFabs using libraries from donors 951 and 1037. In this case the VEEV TC-83 used for panning and screening was inactivated with 0.05% BPL; previously the virus used for these procedures was inactivated with 0.3% BPL, a concentration subsequently shown to reduce the binding of F5 eIgG and F5 nIgG (Table S2). Panning was done with and without epitope masking with non-neutralizing hMAbs F2 eIgG and H6 eIgG in order to increase the probability of isolating Fabs with neutralizing ability. Nine new hFabs were isolated, four using the epitope masking protocol (K1B11, K1H3, L1A7, and K2E2) and five without masking (LR3H11, KR2A3, KR2B12, KR2C2, and 951-D3).

### Antibody binding characteristics and sequence analysis of heavy and light chain complementary determining region 3

HMAbs were titrated on VEEV TC-83 by ELISA (Figure S4). The three hMAbs and mMAb 3B4C-4 had similar binding affinities for TC-83, with titers of approximately 3 ng/ml. All the hFabs had endpoint titers of 5–20 ng/ml with the exception of L1A7 which had a titer of approximately 0.5 µg/ml (Fig. S5).

The viral protein specificities of the hMAbs and hFabs were determined by immunoblot using purified VEEV TC-83 separated by PAGE under both reducing and non-reducing conditions. Seven of 11 antibodies were specific for the E2 glycoprotein and four for E1. All the E2-specific antibodies recognized both reduced and non-reduced protein, whereas the E1-specific antibodies lost reactivity after proteins were subjected to reducing conditions (exposure to 2-mercaptoethanol) (Table 1).

The human antibodies were evaluated by ELISA to determine cross-reactivity with nine VEEVs, representing four subtype 1 varieties as well as subtypes 2–6 (Table 1). HMAb epitope designations were based on the number and specificity of the VEEV subtypes and varieties recognized by each antibody in ELISA and the sequence data for MAb and Fab HC and LC complementary determining region 3 (HCDR3, LCDR3) (Tables 1,2). The cross-reactivity analysis is also presented in graphica

**Table 1:** Characterization of human MAbs and Fabs for Venezuelan equine encephalitis virus (VEEV).

Epitope	µg MAb	MAb^A^	1AB^B^	1C	1D	1E	2	3	4	5	6	PRNT^C^	Immunoblot^D^
hE2^a1^	1	H6 eIgG	++	++	++	++	++	++	−	++	++	>10	+/not tested
hE2^a2^	1	LR3H11	++	++	++	++	++	+	−	+	+	>100	+/+
hE2^b^	1	KR2A3	++	++	++	+	++	+	−	+	+	>100	+/+
hE2^b^	1	KR2B12	++	++	++	+	++	+	−	+	+	>100	+/+
hE2^b^	1	KR2C2	++	++	++	++	++	+	−	+	+	>100	+/+
hE2^c^	1	F5 nIgG	++	++	++	++	++	++	−	−	++	0.01	+/+
hE2^d^	10	951 D3	++	+	+	++	−	−	−	−	−	>100	+/+
mE2^c^	100	Hy4 IgG	++	++	+	−	++	−	−	−	−	<.004	+/+
hE1^a^	1	K1B11^E^	++	++	++	++	++	++	+	++	++	>100	+/−
hE1^a^	1	K1H3^E^	++	++	++	++	++	++	+	++	++	>100	+/−
hE1^b^	10	L1A7^E^	++	++	++	−	+	++	−	+	−	2–3.1	+/−
hE1^c^	1	K2E2^E^	++	++	++	−	++	++	++	++	++	>100	+/−

A MAb tested as full antibody (H6, F5, and Hy4) or Fab. Hy4 IgG is a humanized mouse (m) MAb; all other antibodies are human (h).

B Cross-reactivity reported as percent of each MAb's reactivity with subtype 1AB (VEEV TC-83); ++ (>50%), + (25–50%) and − (<25%). Viruses used for each subtype: 1C (P676), 1D (3880), 1E (Mena II), 2 (Everglades), 3 (Mucambo), 4 (Pixuna), 5 (Cabassou), and 6 (AG80-663).

C Antibody endpoint concentration (µg/ml) in a 70% plaque-reduction neutralization test (PRNT) with VEEV TC-83.

D Antibody reactivity by immunoblot on nonreduced/reduced VEEV TC-83 antigens.

**Figure 1:**
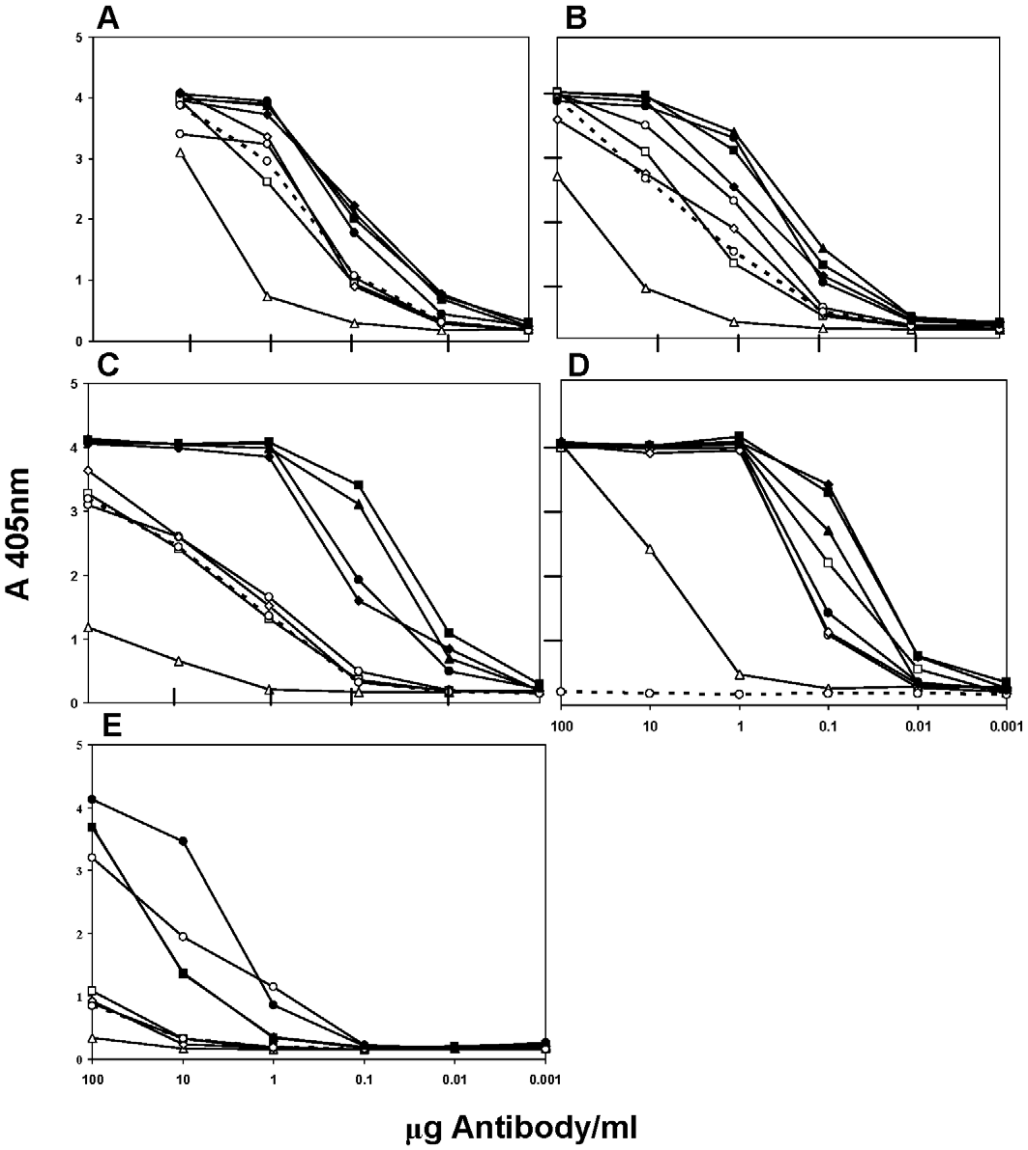
Representative ELISA cross-reactivity patterns for Venezuelan equine encephalitis virus (VEEV) E2-specific human (h) Fabs and MAbs. A. Anti-hE2^a1^ MAb. B. Anti-hE2^a2^ Fab. C. Anti-hE2^b^ Fab. D. Anti-hE2^c^ MAb. E. Anti-hE2^d^ Fab. Four varieties of VEEV subtype 1, TC-83 (1AB, –•–), P676 (1C, –▪–), 3880 (1D, –▴–), and Mena II (1E, –○–); and five other subtypes, EVE (2, –♦–), MUC (3, –□–), PIX (4, –▵–), CAB (5, --○--), and AG80-663 (6, –◊–) were included in the panel of ELISA antigens.

**Figure 2:**
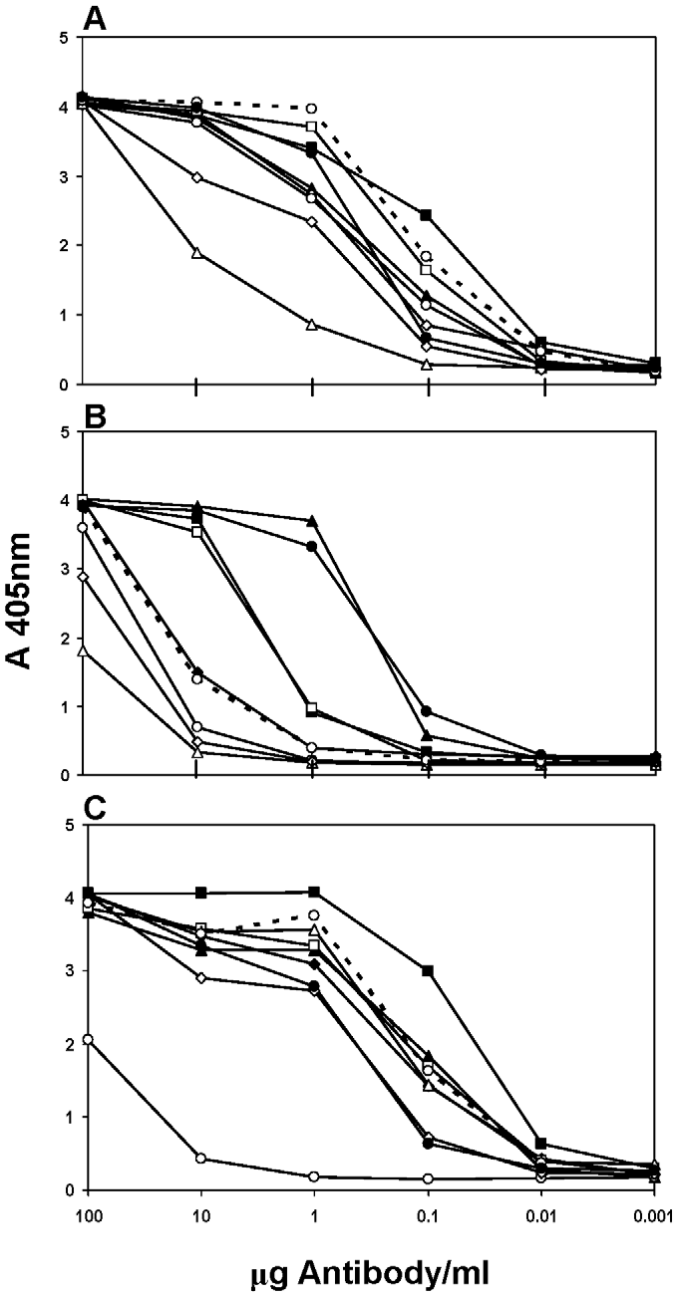
Representative ELISA cross-reactivity patterns for Venezuelan equine encephalitis virus (VEEV) E1-specific human (h) Fabs. A. Anti-hE1^a^. B. Anti-hE1^b^. C. Anti-hE1^c^. Four varieties of VEEV subtype 1, TC-83 (1AB, –•–), P676 (1C, –▪–), 3880 (1D, –▴–), and Mena II (1E, –○–); and five other subtypes, EVE (2, –♦–), MUC (3, –□–), PIX (4, –▵–), CAB (5, --○--), and AG80-663 (6, –◊–) were included in the panel of ELISA antigens.

E Fabs isolated using epitope masking with anti-E2 hMAbs F2 eIgG and H6 eIgG during the panning process.

l form since antibodies with similar cross-reactivities had unique titration curves when antibody concentration was plotted versus absorbance at 405 nm (Figures 1, 2). Sufficient amounts of MAbs F2 eIgG and G1 eIgG were not available to determine ELISA cross-reactivity but each was shown to be specific for E2 by immunoblot and to have PRNT endpoints of ≥10µg/ml (data not shown).

**Table 2:** Amino acid sequences of both heavy and light chain complementary determining region 3 of human MAbs and Fabs for Venezuelan equine encephalitis virus.

Epitope	Type^A^	Antibody	HCDR3^B^	LCDR3^C^
ND^D^	eIgG	F2	QLWFGELFGHDVFDI	QQYHNWPPLT
ND	eIgG	G1	QLWFGELFGHDVFDI	AAWDDSLNGPV
hE2^a1^	eIgG	H6	QLWFGELFGHDVFDI	QVWDSSSDHVV
hE2^a2^	Fab	LR3H11	DTDPFAVLVLAATPADY	CSYAQRFTWV
hE2^b^	Fab	KR2A3	DTDPFAVLVLAATPADY	QQFNDYPAT
hE2^b^	Fab	KR2B12	DTDPFAVLVLAATPADY	QQFNDYPAT
hE2^b^	Fab	KR2C2	DTDPFAVLVLAATPADY	QQANSFPLS
hE2^c^	eIgG, nIgG	F5	DGAYYYDYSGYPYDYNGIDV	AAWDDSLNGWV
hE2^d^	Fab	951 D3	DGGLSEYNYYYYYMDV	QQYYHSPPT
hE1^a^	Fab	K1H3^E^	VKCSSTSCYPWDYYGMDV	QQYNNYPVT
hE1^a^	Fab	K1B11^E^	VKCSSTSCYPWDYYGMDV	QQYNTYPWT
hE1^b^	Fab	K2E2^E^	EENSGYDY	QQSYTTPQYT
hE1^c^	Fab	L1A7^E^	DGAYYYDDSGYPYSYSGIDV	AAWDDSLNGWV

A Antibody used in the form of engineered (e) human IgG, native (n) human IgG or Fab.

B HCDR3, heavy chain complementary determining region 3; amino acid sequence.

C LCDR3, light chain complementary determining region 3; amino acid sequence.

D ND, not done.

E Fabs isolated using epitope masking with anti-E2 MAbs F2 eIgG and H6 eIgG during the panning process.

H6 eIgG, assigned to epitope hE2^a1^, was the most cross-reactive of the anti-E2 MAbs characterized, reacting with all the VEEV subtypes and varieties tested except subtype 4 (Table 1, Figure 1). MAbs F2, G1, and H6 had the same HCDR3 aa sequence, but different LCDR3 sequences, which might indicate that these MAbs bind to different, but overlapping epitopes (Table 2). Fab LR3H11 was assigned to epitope hE2^a2^ based on the similarities of its ELISA titration curve to that for the anti-hE2^a1^ MAb (Figure 1) and its cross-reactivity (Table 1), and on differences in HCDR3 and LCDR3 sequences (Table 2). LR3H11 had a generally lower level of reactivity to all the VEEV subtypes (especially subtypes 3, 5, and 6), compared to H6 eIgG. The higher reactivity level of H6 eIgG compared to Fab LR3H11 could be due to the fact that H6 is a complete IgG molecule. Although the HCDR3 sequence is the same for LR3H11 and the anti-hE2^b^ Fabs, the LCDR3 sequences are completely different (Table 2). The three anti-hE2^b^ Fabs, KR2A3, KR2B12, and KR2C2, have identical HCDR3 sequences and the LCDR3 sequences are 44 to 100% similar (Table 2). Fabs KR2A3 and KR2B12 have the same sequence throughout except for one aa change in framework region 2 (Lys to Arg). In addition, this group of Fabs had a distinct reactivity profile consisting of three reactivity groups (curves) which included the following subtypes and varieties: (1) 1AB, 1C, 1D, 2; (2) 1E, 3, 5, 6; and (3) 4 (Figure 1). MAb F5 nIgG had a unique cross-reactivity pattern and unique HCDR3/LCDR3 sequences as well as potent viral neutralizing activity, and was assigned to epitope hE2^c^ (Tables 1, 2; Figure 1). Fab 951 D3, which was isolated from a different bone marrow donor, also had unique HCDR3/LCDR3 sequences and a unique cross-reactivity pattern, and was assigned to epitope hE2^d^ (Tables 1,2; Figure 1).

The E1-specific hFabs were isolated using epitope blocking during the panning process. Blocking with two E2-specific, non-neutralizing MAbs (F2 and H6) did not have the desired effect of increasing the isolation of neutralizing Fabs; instead this blocking seemed to have inhibited binding of E2-specific Fabs since only E1-specific Fabs were isolated. L1A7 had very limited neutralizing ability, typical for an E1-specific MAb (Table 1). Fabs K1H3 and K1B11 had identical HCDR3 sequences and very similar LCDR3 sequences (two aa differences); these Fabs were assigned to epitope hE1^a^ (Tables 1, 2; Figure 2). Anti-hE1^a^ Fabs were the most cross-reactive antibodies identified, showing good reactivity with all the VEEV subtypes tested. Fabs L1A7 and K2E2 had unique HCDR3 and LCDR3 sequences as well as cross-reactivity patterns, and were assigned to epitopes hE1^b^ and hE1^c^, respectively (Tables 1,2; Figure 2).

### Competitive binding analysis of hMAbs and hFabs

CBAs were performed by ELISA, using VEEV TC-83 as the antigen, to determine if there was spatial overlap between epitope hE2^c^, defined by the neutralizing hMAb F5 nIgG, and the epitopes defined by the other hFabs. Only the homologous F5 Fab or F5 nIgG was able to compete with F5 nIgG, unconjugated or conjugated to HRP (Table 3). None of the anti-hE2^a2^, -hE2^b^, -hE2^d^, -hE1^a^, -hE1^b^, or -hE1^c^ Fabs showed >50% competition with F5 nIgG.

**Table 3:** ELISA-based competition of human Fabs with MAb F5 nIgG for binding to Venezuelan equine encephalitis virus TC-83.

Competitor Fab, IgG	Epitope^A^	F5 nIgG-HRP^B^	Unconjugated F5 nIgG^B^
F5 nIgG	hE2^c^	**92**	Not done
F5 Fab	hE2^c^	**91**	**63**
LR3H11 Fab	hE2^a2^	26	6
KR2B12 Fab	hE2^b^	38	4
KR2C2 Fab	hE2^b^	38	0
951D3 Fab	hE2^d^	35	0
K2E2 Fab	hE1^c^	38	18
K1H3 Fab	hE1^a^	26	0
L1A7 Fab	hE1^b^	36	0

A Epitopes based on cross-reactivity data (see Table 2.); human (h).

B Percent competition values ≥50% were considered significant and are shown in bold font; HRP (horseradish peroxidase).

C HRP, horseradish peroxidase.

CBAs between mMAbs and hMAb F5 were also used to evaluate the spatial similarity of the hE2^c^ epitope defined by MAb F5 nIgG to the previously characterized VEEV E2 epitopes defined by mMAbs which comprise the major E2 neutralization domain (E2 aa182–207) [33], [34]; competition values >50% were considered significant (Table 4). The results showed that epitope hE2^c^ is most spatially aligned with mMAb-defined epitopes E2^d^ and E2^f^; antibodies defining these epitopes showed reciprocal or two-way competition. One-way competitions included F5 nIgG competing with anti-mE2^a^-HRP and anti-mE2^e^-HRP (59% and 56%, respectively); however, neither anti-mE2^a^ nor anti-mE2^e^ could compete with F5-HRP. Murine and humanized anti-E2^c^ MAbs 1A4A-1 and Hy4-IgG [43] competed with F5-HRP (92% and 95%, respectively), but F5 did not significantly block the virus binding of anti-E2^c^ 1A4A-1-HRP (20%). Additionally, there was no competition between F5 and anti-E2^g^ or anti-E2^h^ mMAbs. These analyses indicate that F5 nIgG does not bind to an epitope within the major neutralization domain on the VEEV E2 glycoprotein defined by mMAbs [33].

**Table 4:** Murine and human MAb competition with MAb-hydrogen peroxidase (HRP) conjugates binding to Venezuelan equine encephalitis virus TC-83.

		HRP	HRP	HRP	HRP	HRP	HRP	HRP	HRP	HRP
Competitor^A^	Epitope^B^	α-E2^a^	α-E2^c^	α-E2^d^	α-E2^e^	α-E2^f^	α-E2^g^	α-E2^h^	α-E1^b^	α-hE2^c^
5B4D-6	E2^a^	**88** C								1
1A4A-1	E2^c^		**94**							**92**
1A6C-3	E2^d^			**92**						**60**
1A3A-5	E2^e^				**91**					0
1A4D-1	E2^f^					**90**				**83**
1A3A-9	E2^g^						**94**			0
1A3B-7	E2^h^							**94**		0
3B2A-9	E1^b^								**86**	0
F5 nIgG	hE2^c^	**59**	20	**82**	**56**	**68**	0	30	8	**92**
Hy4 IgG^D^	E2^c^	**59**	**93**	**96**	**90**	**93**	**52**	**95**	30	**95**

A Unlabeled competitor MAb is added to ELISA plate 30 min prior to addition of MAb-HRP conjugates.

B Murine epitopes defined in [34]; h (human) epitope.

C Homologous competition controls are underscored; competition percentages ≥50% were considered significant and are shown in bold font.

D Hy4 IgG is a humanized murine MAb with the specificity of the original anti-E2^c^ MAb [43].

### Reactivity of F5 nIgG with four anti-VEEV mMAb neutralization-escape variants

Anti-VEEV mMAb neutralization-escape variants with mutations in epitopes E2^c^, E2^f^, E2^g^, or E2^h^ were evaluated for reactivity with F5 nIgG [36]. If the epitope recognized by F5 were the same as, or near, any of the changed aa residues (E2-182, 183, 199, and 207) in the neutralization-escape variant viruses, then F5 would be unable to neutralize that variant. Results indicated that F5 was able to neutralize all the variant viruses, including v3B2A-9 which is specific for an E1 epitope (Table 5). F5 nIgG PRNT endpoints were equivalent for all the variant viruses as well as the parent VEEV TC-83.

**Table 5:** Neutralization of five murine MAb neutralization-escape variants of Venezuelan equine encephalitis virus (VEEV) TC-83 by human MAb F5 nIgG.

			70% PRNT endpoint^D^	70% PRNT endpoint
Virus/variant^A^	Epitope^B^	Variant aa^C^	F5 nIgG ng/ml	Homologous mMAb µg/ml
VEEV TC-83	—	—	6.25	—
v3B4C-4	E2^c^	E2 aa 182	12.5	>100
v1A4D-1	E2^f^	E2 aa 183	12.5	≥100
v1A3A-9	E2^g^	E2 aa 199	6.25	50
v1A3B-7	E2^h^	E2 aa 207	6.25	100
v3B2A-9	E1^b^	—	6.25	<1∶100 (MAb ascites)

A Variant (v) virus designation is the name of the MAb used to generate each variant [36].

B Epitope designation for each MAb used to create the neutralization-escape variant virus.

C Location of the E2 protein amino acid (aa) change for each variant.

D PRNT, plaque-reduction neutralization test; a 70% PRNT endpoint is given in ng/ml for F5 and µg/ml for each homologous murine (m) MAb.

### Selection of F5 nIgG neutralization-escape variant viruses

MAb F5 nIgG was used to select neutralization-escape variants of the VEEV TC-83 infectious clone, pVE/IC-92, in order to more accurately map the binding site of this potent neutralizing antibody. Two variant (v) viruses were isolated, vF5 nIgG-3 (vF5-3) and vF5 nIgG-5 (vF5-5), which required either 500- or 2000-fold more F5 nIgG for neutralization than that needed for neutralization of parental VEEV TC-83 (Table 6). Neutralization of these variants was also evaluated with the humanized Hy4 IgG which is known to bind to the mE2^c^ epitope (E2 aa182) [36]. The vF5-5 virus required ≥4000-fold more Hy4 IgG than the parent virus for neutralization, and thus was neutralization-resistant for both F5 nIgG and Hy4 IgG. Although the vF5-3 virus resisted neutralization with F5 nIgG, Hy4 neutralized this variant as well as it neutralized VEEV TC-83.

**Table 6:** Comparison of the neutralization activity of humanized murine MAb Hy4 IgG and fully human MAb F5 nIgG for parental Venezuelan equine encephalitis virus (VEEV) TC-83 and F5 nIgG neutralization-escape variant viruses vF5 nIgG-3 and vF5 nIgG-5.

Virus, variant (v)	F5-PRNT-1^a^	F5-PRNT-2^a^	Hy4-PRNT-1^b^	Hy4-PRNT-2^b^
vF5 nIgG-3	6.25 (500)^c^	6.25 (500)	<0.05 (<2)	0.047 (3.75)
vF5 nIgG-5	25 (2000)	18.8 (1500)	>100 (>4000)	50 (4000)
VEEV TC-83	0.0125	0.0125	0.025	0.0125

a PRNT (plaque-reduction neutralization test) using F5 nIgG; 70% PRNT endpoints are reported in µg/ml of MAb.

b PRNT using Hy4 IgG; 70% PRNT endpoints are reported in µg/ml of MAb.

c Fold difference in PRNT endpoints between variant and parental VEEV TC-83 are shown in parentheses.

Genomes of the variant and parental VE/IC-92 viruses were sequenced from nucleotide 8195 to 11421, which includes genetic information for viral structural proteins E1, E2, E3, 6K and a portion of the capsid. Mutations in the variant viruses were found only in the E2 glycoprotein between aa 115–119 (Table 7). The vF5-3 virus had a transition from A to G at nucleotide 8906, resulting in a non-conservative amino acid change from Lys to Glu at E2 aa115. There was also a deletion of nucleotides 8918–8923, coding for Val (E2 aa119), in the parent virus. The vF5-5 virus had a silent G to A change at nucleotide 8908, followed by a deletion of nucleotides 8909–8911, which code for Lys (E2 aa116). The sequence change at E2 aa115 and deletions at E2 aa116 and 119 in these neutralization-escape variant viruses indicate that F5 binds at this site in the E2 glycoprotein. F5 nIgG neutralizing activity was somewhat more reduced with vF5-5 virus than with vF5-3, suggesting that the deletion of Lys is more disruptive for F5 binding than the Lys to Glu substitution coupled with the Val deletion found in the vF5-3 virus.

**Table 7:** Sequence comparisons between Venezuelan equine encephalitis virus TC-83 (VE/IC-92) and two F5 nIgG neutralization-escape variant viruses vF5 nIgG-3 and vF5 nIgG-5.

		Nucleotide sequence (nt 8906–8923)
Virus, variant (v)	nt/aa	E2 amino acid sequence (aa 115–120)
TC-83 (VE/IC-92)	nt	AAG AAA GAT TCC GTC AGA
	aa	K K D S V R
vF5 nIgG-3	nt	**G**AG AAA GAT TCC **---** a AGA
	aa	**E** K D S R
vF5 nIgG-5	nt	AA**A** **---** GAT TCC GTC AGA
	aa	K D S V R

a ---, deleted sequence.

## Discussion

The humoral immune response to VEEV infection in mice has been well characterized and anti-VEEV mMAbs have played an essential role in identifying the important antigenic domains of the virus glycoproteins [33]–[35]. Although non-human primate models have been used to evaluate vaccine efficacy of candidate VEEVs, the antibody repertoires in these primates and humans have not been well characterized [16], [17], [53]. Use of phage antibody libraries generated from immune or nonimmune donors has proved to be an efficient method for obtaining a diverse set of non-human primate or human antibodies for a wide variety of viruses: VEEV [54], human papillomavirus [55], Sin Nombre virus [56], West Nile virus [57], yellow fever virus (YFV) [58], rabies virus [59], SARS-coronavirus [60], hepatitis A virus (HAV) [61], dengue 4 virus (DENV4) [62], rotavirus [63], Hantaan virus [64], measles virus [65], and HIV [66]. In this study we have characterized 11 unique hFabs and hMAbs for viral protein specificity, VEEV subtype cross-reactivity, and virus neutralization capacity and constructed the first human epitope map for the E1 and E2 proteins of an alphavirus. In addition we have mapped the potential E2 binding site of the potent neutralizing hMAb F5 nIgG using CBAs with the other hFabs as well as anti-VEEV mMAbs, and isolated and sequenced the RNA coding for E1 and E2 glycoproteins of F5 neutralization-escape variant viruses.

The antibody clones selected for characterization showed good ELISA binding to VEEV TC-83 and had unique complete gene sequences; seven hMAbs were specific for the E2 glycoprotein and four for E1. Only two neutralizing hFabs, F5 and L1A7, were isolated, despite the use of a blocking strategy during panning to favor selection of such antibodies. A similar strategy was used, without much success, in an attempt to favor selection of West Nile virus hMAbs specific for domain III of the envelope glycoprotein [57]. The anti-E2 specific F5 nIgG had a 70% PRNT endpoint of 10 ng/ml, equivalent to that described for the most effective neutralizing anti-VEEV E2 mMAbs (Table 1) [33], [34]. Poorer neutralization titers for human or chimpanzee Fabs or MAbs specific for YFV, DENV4 or HAV have been reported: 0.1–3 µg/ml, 0.2–0.6 µg/ml, and 0.5 µg/ml, respectively [58], [61], [62]. The E1-specific hFab L1A7 had a PRNT endpoint of 3 µg/ml, 300-fold lower than F5. The titer might be higher for a complete IgG antibody, but typically neutralization titers for anti-E1 mMAbs are less than anti-E2 mMAbs [34].

A high degree of cross-reactivity for the various VEEV subtypes and varieties was found for the E2-specific hMAbs and hFabs compared to the spectrum of type-specific to subgroup-reactive reactivities shown by the well-characterized mMAbs (Table 1) [33], [34], [67]. In fact, no VEEV type-specific hMAbs were isolated. The methodology for generating the mMAbs was different from that used in this study, but in all studies screening was done on plate-bound, purified virus and clones were selected that had the highest level of binding by ELISA. The virus used for panning in this study was inactivated with either 0.3% or 0.05% BPL, while live virus was used in ELISAs to select mMAbs. However, the BPL-treated virus was reactive with neutralizing mMAbs in ELISA and it was assumed that neutralizing hMAbs would also bind this antigen. HMAb F5 nIgG was originally selected using 0.3% BPL-treated virus, but it was later discovered that its binding to live virus was 50–75% greater (Table S2); therefore, the amount of BPL used was decreased to 0.05%, which improved F5 binding (data not shown). The fact that the binding of mMAbs was not affected by treating virus with 0.3% BPL, but binding of hMAb F5 was affected, suggests that epitopes recognized by the VEEV neutralizing mMAbs and hMAb F5 may not be the same.

The ELISA binding affinity of the three human IgG antibodies, F5, H6, and G1, for VEEV TC-83 was equivalent to that of mMAb 3B4C-4 (Figure S4). Both F5 nIgG and 3B4C-4 are potent neutralizing MAbs while neither H6 eIgG nor G1eIgG have biological activity, so in this case high affinity was not necessarily correlated with neutralization capacity. CBA analysis indicated that the hE2^c^ epitope defined by F5 nIgG does not spatially overlap any of the other epitopes defined by the panel of hFabs (Table 3). The cross-reactivity profile of this hMAb was also unique (Table 1, Figure 1). CBA results comparing F5 and a panel of mMAbs important in defining a major VEEV E2 neutralization domain suggested that epitopes mE2^d^ and mE2^f^ were spatially near epitope hE2^c^ based on reciprocal competition patterns (Table 4). The mE2^f^ epitope has been mapped to residue E2-183, but mE2^d^ has not yet been mapped [36]. However, in the original description of this neutralization domain defined by mMAbs, there was no direct competition between mE2^d^ and mE2^f^ [33]. Further analysis revealed that F5 neutralized all four mMAb neutralization-escape variant viruses to the same degree as the parent VEEV TC-83, indicating that binding of F5 was not affected by an aa change at either E2-182, -183, -199 or -207, providing important evidence that F5 recognized a different E2 epitope than any of the four neutralizing mMAbs used to generate the variant viruses (Table 5).

We isolated two neutralization-escape variant viruses of F5 nIgG, vF5-3 and vF5-5, and sequenced their structural protein genes to locate the theoretical binding site of this hMAb. Based on the aa changes in the variant viruses, F5 binding was mapped to aa residues E2-115 to 119 (Table 7). The aa deletions and substitution in vF5-5 and vF5-3 viruses alter the net charge or degree of hydrophilicity in this E2 region, possibly affecting the accessibility of this epitope for antibody binding. Variant virus vF5-5 was more resistant than vF5-3 to neutralization with F5, although both variant viruses required significantly more MAb for neutralization than required for VEEV TC-83 (Table 6). We also tested whether or not the humanized mMAb Hy4 IgG, specific for the mE2^c^ epitope, could neutralize these two variant viruses. If the binding sites for F5 and Hy4 are different, it would be expected that Hy4 would neutralize both F5 variant viruses. However, only vF5-3 was neutralized, and vF5-5 was as resistant to neutralization by Hy4 as it was to F5. This rather puzzling result, in addition to the reciprocal competition between F5 and the anti-E2^f^ mMAb, might suggest some type of interaction or induced conformational change between the neutralization domains located at E2 aa182–207 and E2 aa115–119. Of course the possibility cannot be excluded that the detected mutations are not the contact residues for F5, but that these amino acid substitutions or deletions induce distant conformational changes that affect MAb binding.

The identification of a novel neutralization domain on the VEEV E2 glycoprotein is analogous to the identification of a second neutralization domain on SV, identified using anti-E2c MAbs R6 and R13 neutralization-escape variant viruses that contained a coding change at either E2 aa62, 96, or 159 [68]. It was proposed that these E2 residues that formed an alternative neutralization site could be folded to form a binding site with the surface dimensions of approximately 600–750 Å, measurements similar to those determined for the interaction of lysozyme–anti-lysozyme immune complexes [69]. Transposon-insertion mutagenesis of SV resulted in a virus with an insertion at E2-119 that was less efficiently neutralized by SV mMAbs 202 (anti-E2ab) and 209 (anti-E2c) [70]. Similarly, a variant of RRV, attenuated in mice, had five E2 aa differences compared to wild-type at positions 3, 67, 119, 251, and 302 [39]. Residue 251 lies in the major neutralization domain, whereas residues 67 and 119 were proposed to influence neutralization efficiency in the variant virus. Examination of VEEV E2 mutations that affect virus binding to heparan sulfate led to a proposal that E2 residues 76 and 116 may form a conformational, surface-accessible epitope, but its involvement with virus neutralization is unknown [71].

**Figure 3:**
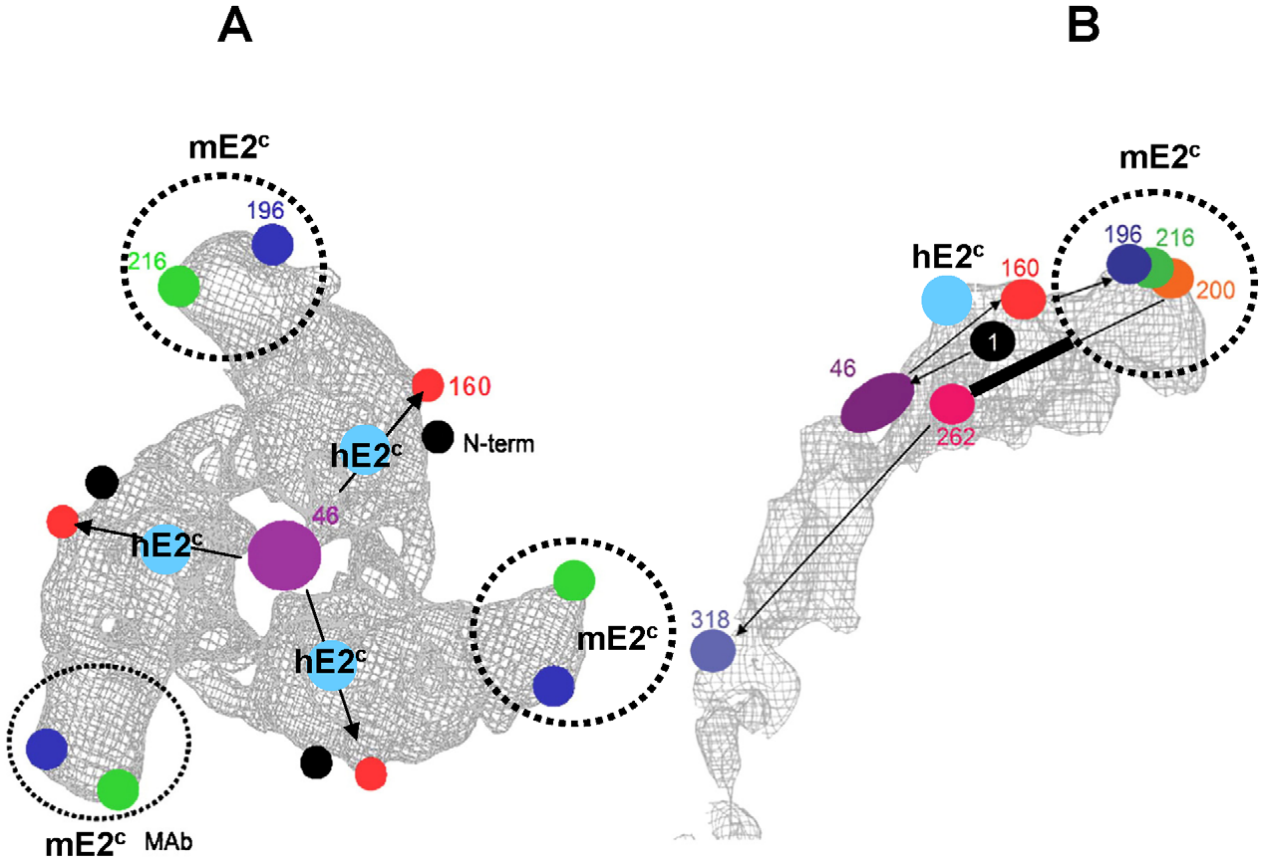
Mapping the E2 ectodomain. A. A top view of the E2 density of one spike looking down the 3‐fold axis (shown as the large purple spot corresponding to three merged carbohydrate moieties at position 46). B. A side view of one E2 molecule with the approximate location of Venezuelan equine encephalitis virus (VEEV) E2 peptide 13 (amino acids 241–265) shown in black. A and B: The markers on the E2 glycoprotein that correspond to carbohydrate moieties at positions 46, 160, 196, 200, 216, 262, and 318 are shown in purple, red, blue, orange, green, pink, and light blue, respectively. Position 216 (green) was also identified with a cryoEM map of a Fab‐Ross River virus complex. Approximate locations of the VEEV murine MAb anti‐mE2^c^ binding site and critical neutralization site are shown by a dotted circle; the proposed binding site of the VEEV human MAb anti‐hE2^c^ is shown in cyan.

Although the crystal structure of the alphavirus E2 glycoprotein has not been solved, cryoEM reconstructions of E2 have been reported [25], [30], [72]. The 9Å resolution cryoEM map of the SV E2 presented by Mukhopadhyay et al. [72] was annotated with markers representing locations of glycosylation sites, the protein N-terminus, and a neutralizing Fab binding site. We have adapted their figure to show the probable surface-accessible location of the hMAb F5 nIgG binding site (E2 aa115–119) and its relationship with other markers (Figure 3A,B). Mapping of this epitope to a unique E2 neutralization site was based on the data presented in this study: (1) epitope binding by hMAb F5 nIgG was more sensitive to 0.3% BPL treatment than epitopes recognized by neutralizing mMAbs, (2) hMAb F5 was able to neutralize all anti-VEEV mMAb neutralization escape variant viruses and therefore did not bind to E2 residues 182–207, defined as the “critical” neutralization domain, and (3) hMAb F5 neutralization escape variant viruses vF5-3 and vF5-5 defined a neutralization epitope involving E2 aa115–119. Results from studies in mice using VEEV E2 synthetic peptides as vaccines have been included in the proposed map of the E2 ectodomain to complement the placement of the hE2^c^ epitope (Figure 3B). Previously, we identified two peptide vaccines, VE2pep01 (E2 aa1–25) and VE2pep13 (E2 aa241–265) that protected mice from virulent VEEV challenge [73]–[75]. We also isolated an anti-peptide MAb, 1A2B-10, specific for E2 aa1–19, which passively protected mice challenged with VEEV varieties 1AB, 1C, and 1D [76]. None of the anti-peptide antibodies, either polyclonal or monoclonal, had virus-neutralizing activity, indicating that their cognate peptides were not likely to be surface-accessible or lacked the appropriate conformation. The proposed configuration of the E2 molecule shown in Fig. 3B places the hE2^c^ epitope (E2 aa115–119) on the surface of the spike above the more cryptic locations of the E2 N-terminus (VE2pep01) and aa 241–265 (VE2pep13). Such an arrangement would be in agreement with the current knowledge of the structure of the E2 glycoprotein, the location of specific markers, and functional attributes of specified epitopes. We are now in collaboration to obtain structural data on Fab-virion complexes to determine actual binding sites of F5 nIgG and other human and murine MAbs.

The VEEV neutralizing ability of the hMAb F5 nIgG is similar to that exhibited by the humanized mMAb Hy4 IgG. When administered prophylactically, as little as 100 ng of Hy4 was able to protect 90% of mice challenged intraperitoneally with virulent VEEV [43]. In addition, Hy4 given one or 24 h after VEEV infection cured 90% or 75% of infected mice, respectively. F5 nIgG would be expected to be as effective an immunotherapeutic as Hy4 IgG. Administration of a cocktail of the two MAbs, which bind to different epitopes, could provide increased protection against generating virulent VEEV neutralization-escape variants *in vivo*.

## Supporting Information

Figure S1 Cloning of Fabs into the expression vector PAEV1.(A) Light and heavy chains of selected Fabs were inserted into vector PAEV1 at Eco RI/Spe I sites. (B) Fabs with light chains containing Eco RI sites were inserted into vector PAEV1 (containing the light and heavy chains of Fab L1A7) at Xba I/Age I sites.
(0.06 MB PPT)

Figure S2 Schematic presentation of conversion of Fabs to engineered IgG (eIgG). A fragment containing mammalian control elements was inserted into each Fab at Not I and Xho I sites in the PAX243 Fab vector. The Sfi I to Age I fragment containing the light chain, mammalian control elements, and the heavy chain was transferred to an IgG expression vector containing mammalian elements upstream of the light chain as well as IgG CH1, hinge, CH2, and CH3 regions downstream from the heavy chain. Final construct of eIgG retained the engineered Xba I, Sac I, and Xho I sites.
(0.02 MB PPT)

Figure S3 Conversion of engineered (e) F5 eIG to native (n) F5 nIgG. This conversion was done in a 3 step process: (1) Hind III in the mammalian control elements for the heavy chain was changed to Asc I by site-specific mutagenesis and overlap PCR. (2) Xba I and Sac I sites from the light chain and the Xho I site from the heavy chain were converted to native sequences by site-specific mutagenesis and overlap PCR. (3) The engineered Xho I site was changed to native sequence by site-specific mutation and overlap PCR.
(0.07 MB PPT)

Figure S4 MAbs binding to native Venezuelan equine encephalitis virus TC-83. Human MAbs: F5 nIgG ♦, H6 eIgG •, and G1eIgG ▴; murine MAb 3B4C-4 ▪.
(0.07 MB PPT)

Figure S5 Binding of purified Fabs to inactivated Venezuelan equine encephalitis virus TC-83. Solid lines are Fab binding to VEEV TC-83: KR2B12, black inverted triangles; KR2A3, pink diamonds; LR3H11, blue triangles; K1B11, magenta circles; KR2C2, black squares; K2E2, green circles; K1H3, orange diamonds; L1A7, cyan squares; and F5, red squares. The black circle/dashed line is a representative Fab (K1B11) binding to the ovalbumin negative antigen control.
(0.07 MB PPT)

Table S1 Primers used in reverse transcription PCR and sequencing reactions.
(0.03 MB DOC)

Table S2 ELISA binding of purified human and murine Venezuelan equine encephalitis virus (VEEV) MAbs to either native or 0.3% β-propiolactone (BPL)-treated VEEV TC-83.
(0.03 MB DOC)
