# Supplementary material for: The First Human Epitope Map of the Alphaviral E1 and E2 Proteins Reveals a New E2 Epitope with Significant Virus Neutralizing Activity
Source: PLoS Negl Trop Dis. 2010 Jul 13;4(7):e739. doi: 10.1371/journal.pntd.0000739 (PMC2903468; doi:10.1371/journal.pntd.0000739)
Supplement: Figure S1 — Cloning of Fabs into the expression vector PAEV1.(A) Light and heavy chains of selected Fabs were inserted into vector PAEV1 at Eco RI/Spe I sites. (B) Fabs with light chains containing Eco RI sites were inserted into vector PAEV1 (containing the light and heavy chains of Fab L1A7) at Xba I/Age I sites. (0.06 MB PPT) [file pntd.0000739.s001.ppt]

## Slide 1
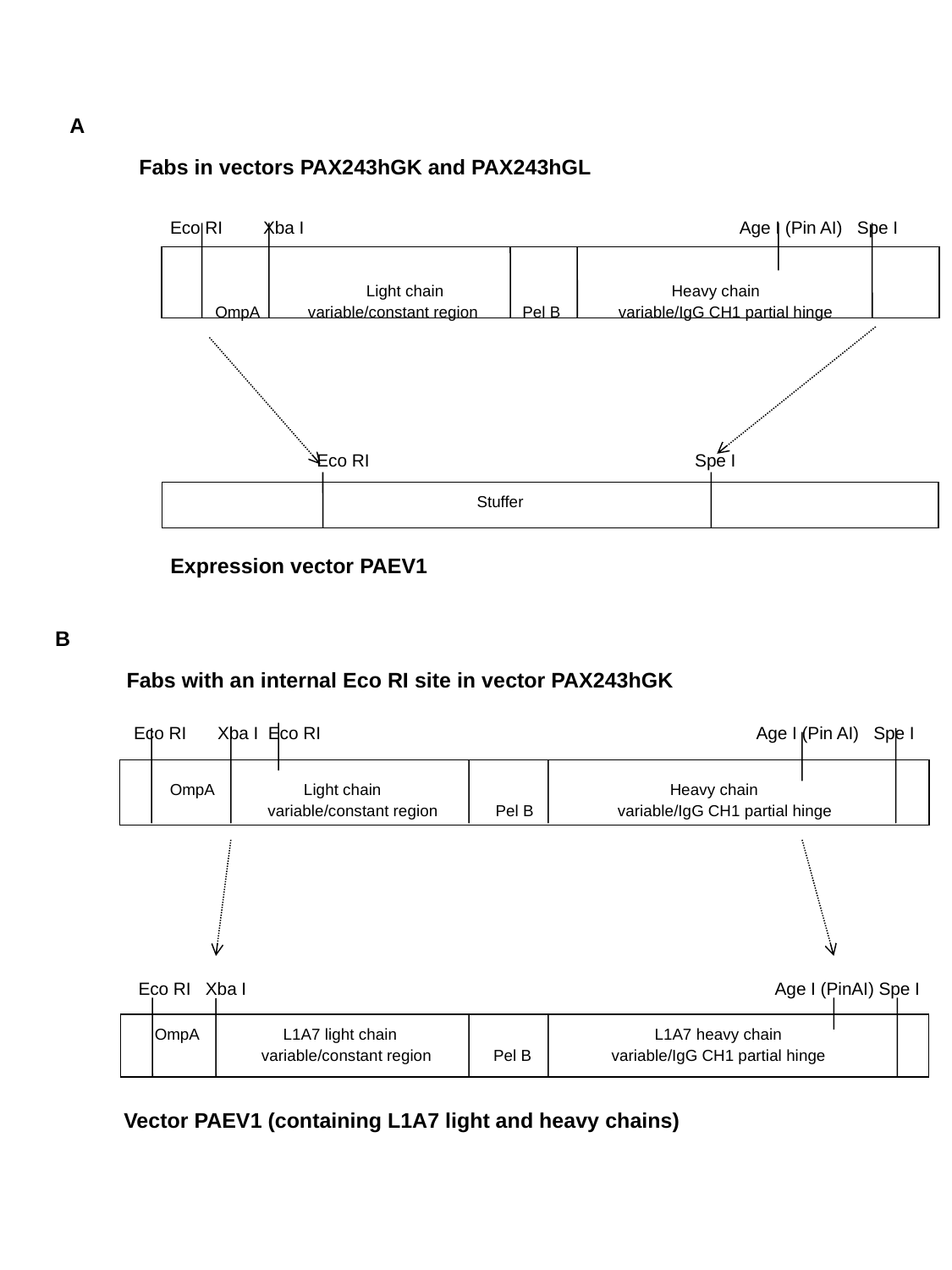

A
 Fabs in vectors PAX243hGK and PAX243hGL
 Eco RI	 Xba I Age I (Pin AI) Spe I
 Light chain	 Heavy chain
 OmpA variable/constant region Pel B	 variable/IgG CH1 partial hinge
 Eco RI Spe I
 Stuffer
 Expression vector PAEV1
B
Fabs with an internal Eco RI site in vector PAX243hGK
 Eco RI Xba I Eco RI Age I (Pin AI) Spe I
 OmpA 	 Light chain Heavy chain
 variable/constant region Pel B	 variable/IgG CH1 partial hinge
 Eco RI Xba I Age I (PinAI) Spe I
 OmpA L1A7 light chain L1A7 heavy chain
 variable/constant region Pel B variable/IgG CH1 partial hinge
Vector PAEV1 (containing L1A7 light and heavy chains)
