# Supplementary material for: The First Human Epitope Map of the Alphaviral E1 and E2 Proteins Reveals a New E2 Epitope with Significant Virus Neutralizing Activity
Source: PLoS Negl Trop Dis. 2010 Jul 13;4(7):e739. doi: 10.1371/journal.pntd.0000739 (PMC2903468; doi:10.1371/journal.pntd.0000739)
Supplement: Figure S2 — Schematic presentation of conversion of Fabs to engineered IgG (eIgG). A fragment containing mammalian control elements was inserted into each Fab at Not I and Xho I sites in the PAX243 Fab vector. The Sfi I to Age I fragment containing the light chain, mammalian control elements, and the heavy chain was transferred to an IgG expression vector containing mammalian elements upstream of the light chain as well as IgG CH1, hinge, CH2, and CH3 regions downstream from the heavy chain. Final construct of eIgG retained the engineered Xba I, Sac I, and Xho I sites. (0.02 MB PPT) [file pntd.0000739.s002.ppt]

## Slide 1
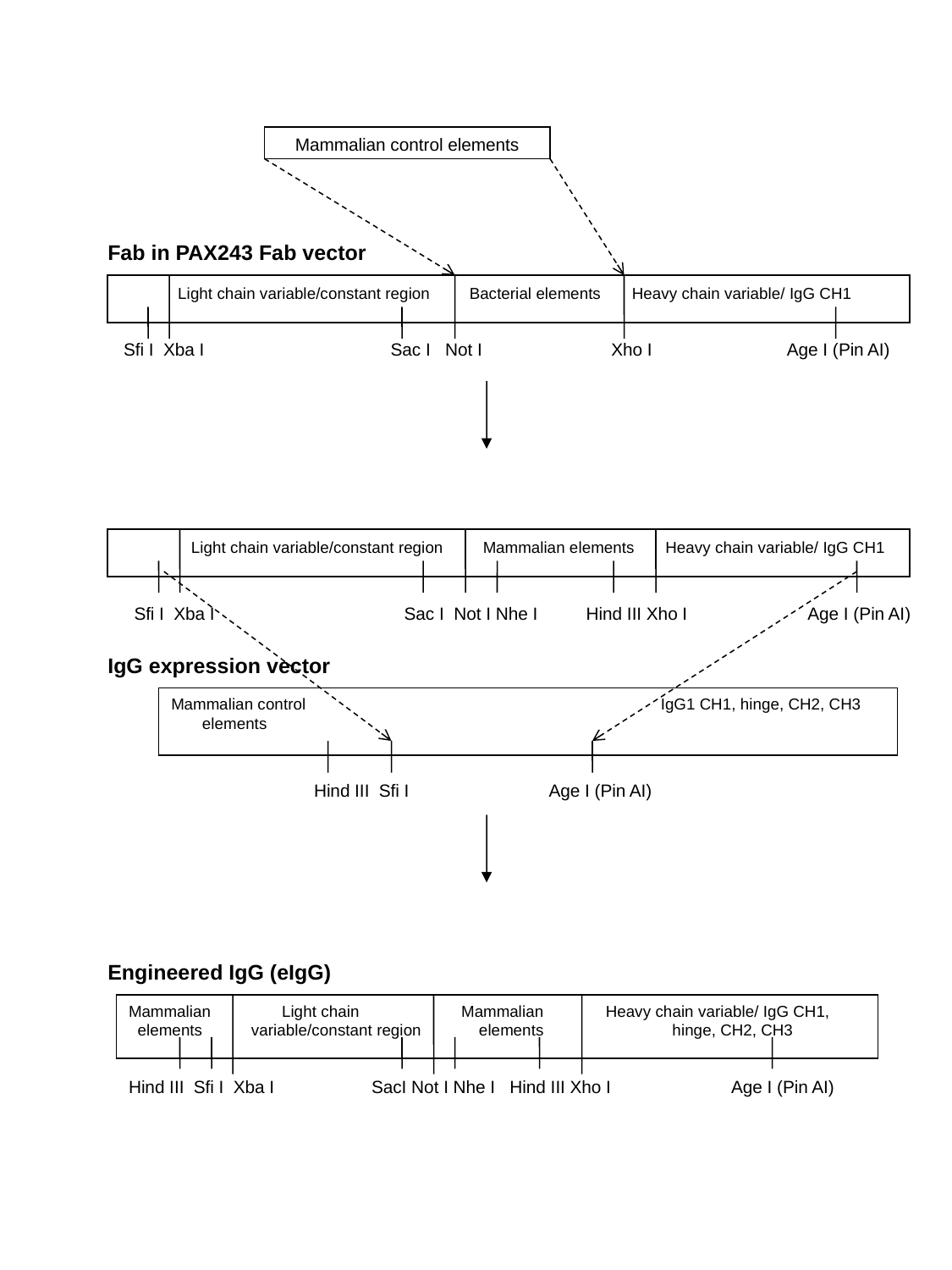

Mammalian control elements
Fab in PAX243 Fab vector
 Light chain variable/constant region Bacterial elements Heavy chain variable/ IgG CH1
 Sfi I Xba I Sac I Not I Xho I Age I (Pin AI)
 Light chain variable/constant region Mammalian elements Heavy chain variable/ IgG CH1
 Sfi I Xba I Sac I Not I Nhe I Hind III Xho I Age I (Pin AI)
IgG expression vector
Mammalian control IgG1 CH1, hinge, CH2, CH3
 elements
Hind III Sfi I Age I (Pin AI)
Engineered IgG (eIgG)
Mammalian Light chain Mammalian Heavy chain variable/ IgG CH1,
 elements variable/constant region elements hinge, CH2, CH3
Hind III Sfi I Xba I SacI Not I Nhe I Hind III Xho I Age I (Pin AI)
