# Supplementary material for: The First Human Epitope Map of the Alphaviral E1 and E2 Proteins Reveals a New E2 Epitope with Significant Virus Neutralizing Activity
Source: PLoS Negl Trop Dis. 2010 Jul 13;4(7):e739. doi: 10.1371/journal.pntd.0000739 (PMC2903468; doi:10.1371/journal.pntd.0000739)
Supplement: Figure S3 — Conversion of engineered (e) F5 eIG to native (n) F5 nIgG. This conversion was done in a 3 step process: (1) Hind III in the mammalian control elements for the heavy chain was changed to Asc I by site-specific mutagenesis and overlap PCR. (2) Xba I and Sac I sites from the light chain and the Xho I site from the heavy chain were converted to native sequences by site-specific mutagenesis and overlap PCR. (3) The engineered Xho I site was changed to native sequence by site-specific mutation and overlap PCR. (0.07 MB PPT) [file pntd.0000739.s003.ppt]

## Slide 1
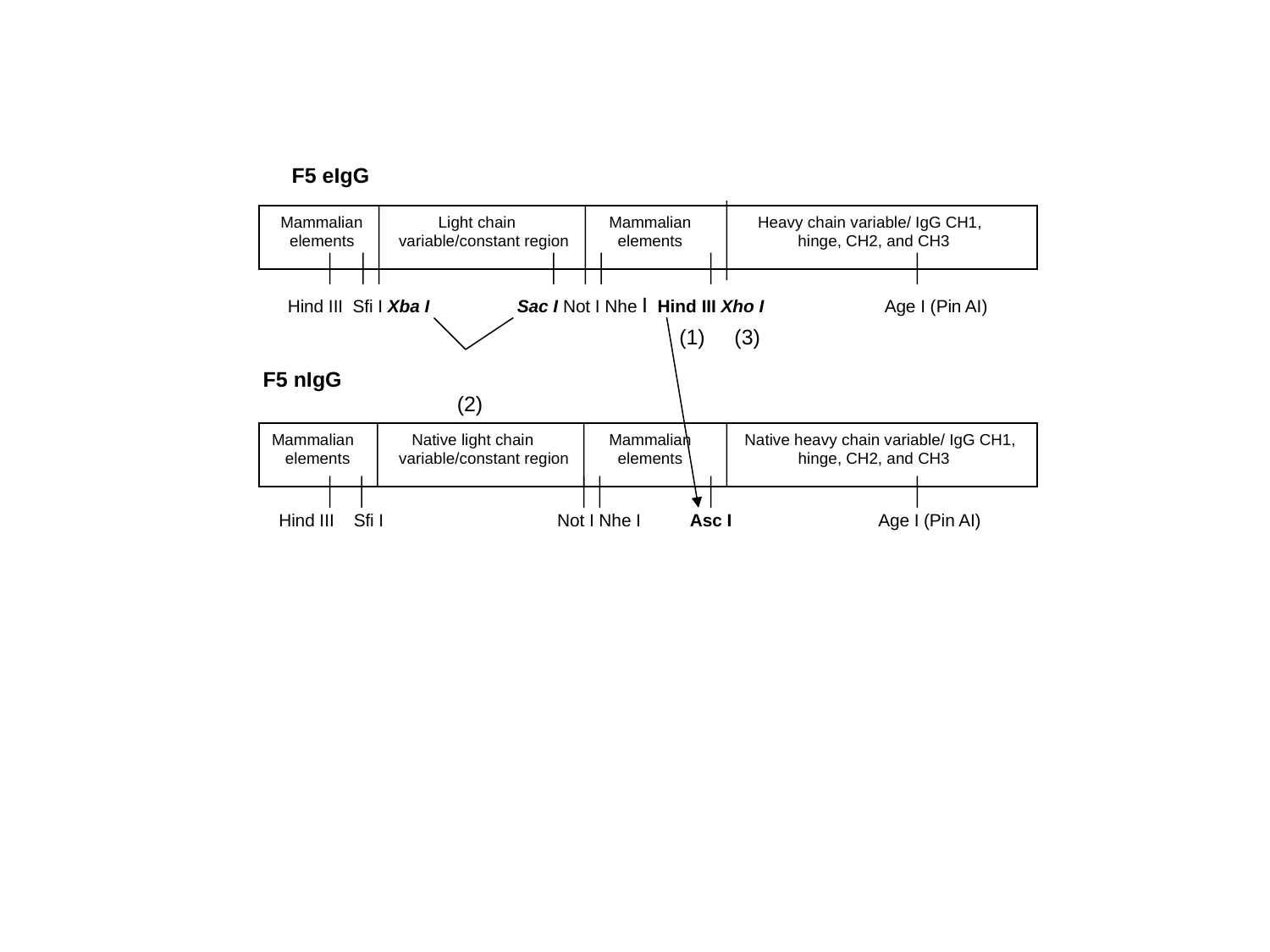

F5 eIgG
 (2)
 Mammalian Light chain Mammalian Heavy chain variable/ IgG CH1,
 elements variable/constant region elements hinge, CH2, and CH3
Hind III Sfi I Xba I Sac I Not I Nhe I Hind III Xho I Age I (Pin AI)
(1) (3)
F5 nIgG
Mammalian Native light chain Mammalian Native heavy chain variable/ IgG CH1,
 elements variable/constant region elements hinge, CH2, and CH3
Hind III Sfi I Not I Nhe I Asc I Age I (Pin AI)
