# Supplementary figures and images for: The First Human Epitope Map of the Alphaviral E1 and E2 Proteins Reveals a New E2 Epitope with Significant Virus Neutralizing Activity
Source: PLoS Negl Trop Dis. 2010 Jul 13;4(7):e739. doi: 10.1371/journal.pntd.0000739 (PMC2903468; doi:10.1371/journal.pntd.0000739)

## Slide 1
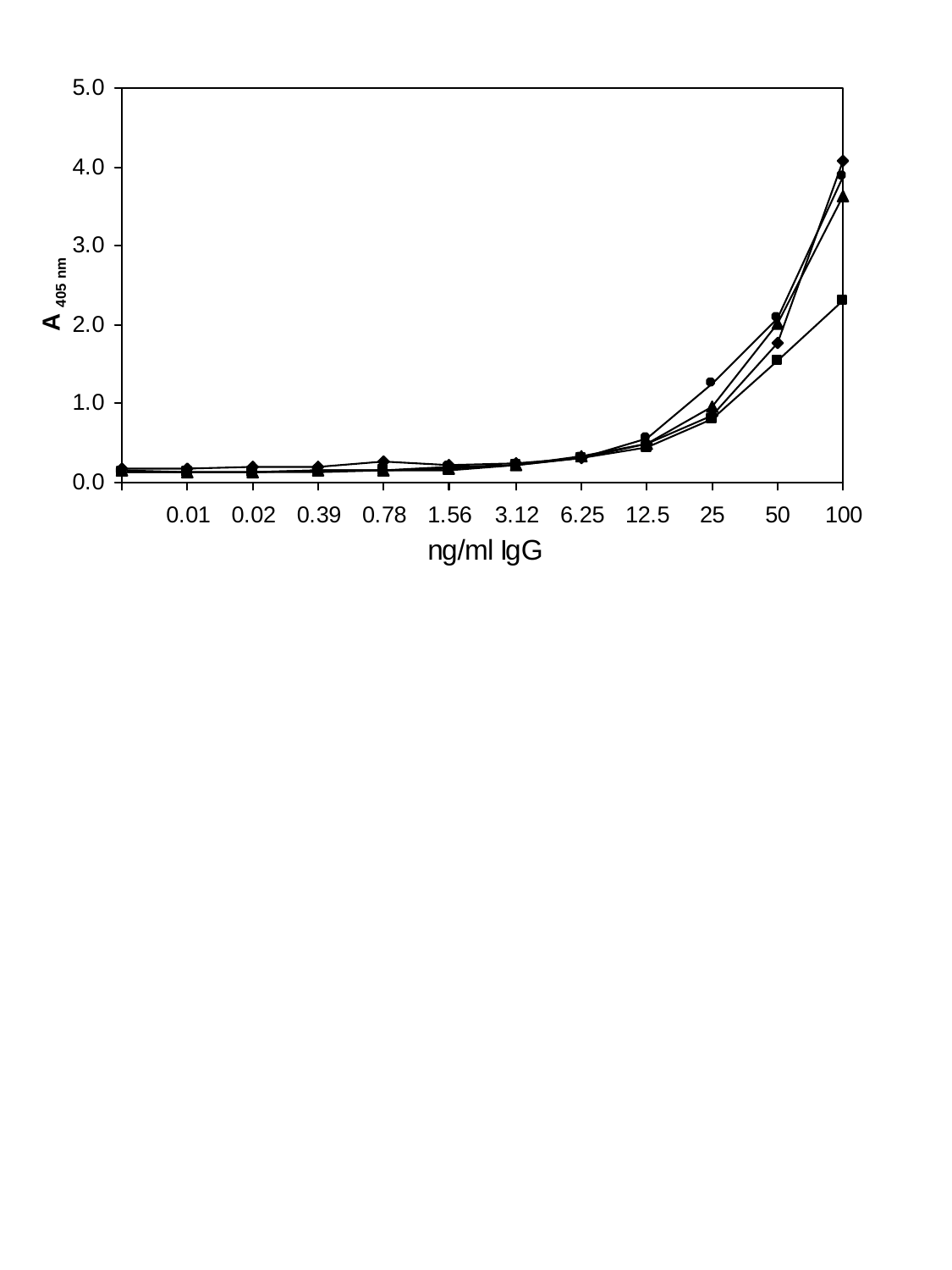

A 405 nm

Supplement: Figure S4 — MAbs binding to native Venezuelan equine encephalitis virus TC-83. Human MAbs: F5 nIgG ♦, H6 eIgG •, and G1eIgG ▴; murine MAb 3B4C-4 ▪. (0.07 MB PPT) [file pntd.0000739.s004.ppt]

## Slide 1
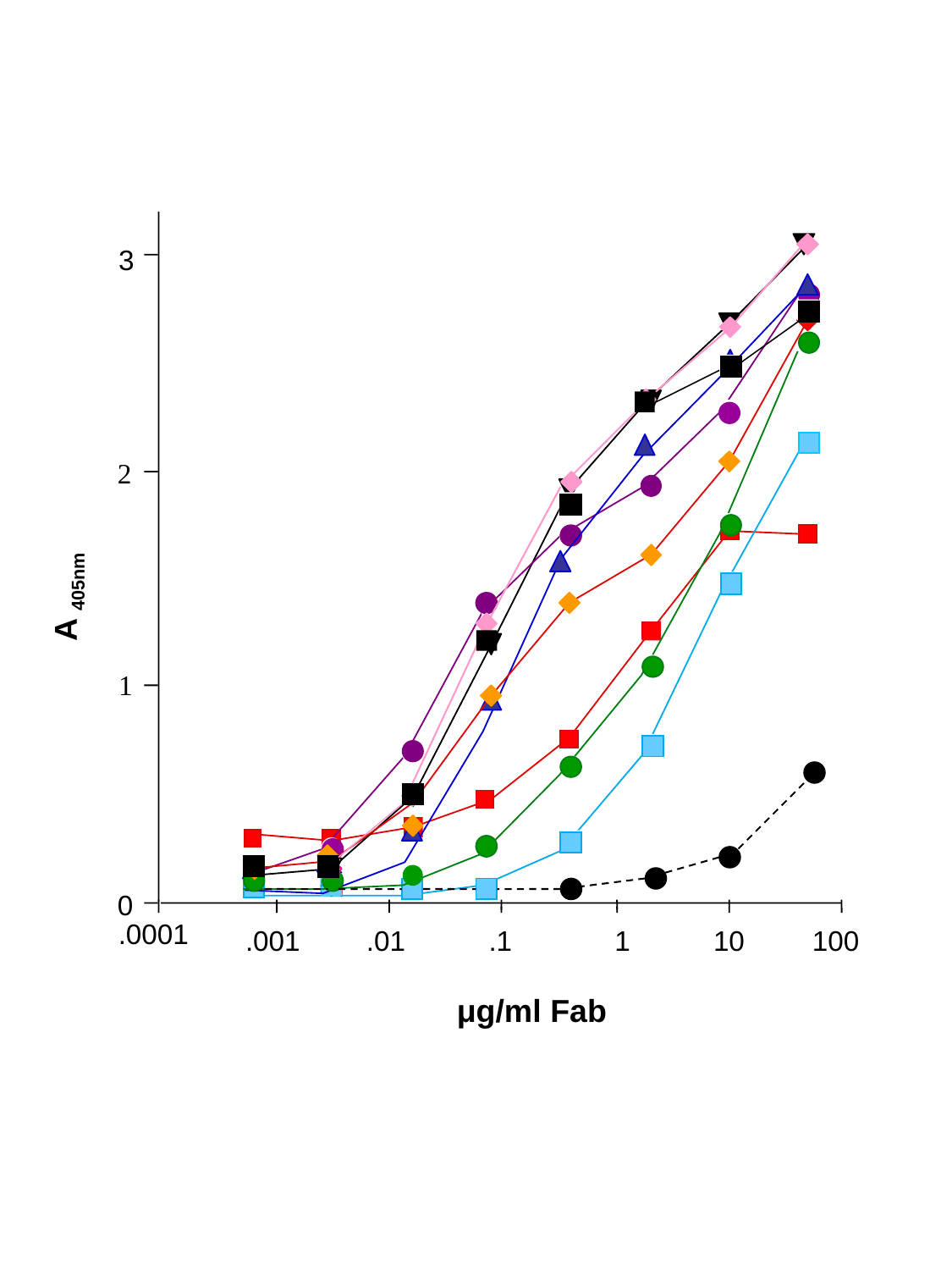

3
2
A 405nm
1
0
.0001
.001
.01
.1
1
10
100
μg/ml Fab

Supplement: Figure S5 — Binding of purified Fabs to inactivated Venezuelan equine encephalitis virus TC-83. Solid lines are Fab binding to VEEV TC-83: KR2B12, black inverted triangles; KR2A3, pink diamonds; LR3H11, blue triangles; K1B11, magenta circles; KR2C2, black squares; K2E2, green circles; K1H3, orange diamonds; L1A7, cyan squares; and F5, red squares. The black circle/dashed line is a representative Fab (K1B11) binding to the ovalbumin negative antigen control. (0.07 MB PPT) [file pntd.0000739.s005.ppt]
