# Supplementary material for: The First Human Epitope Map of the Alphaviral E1 and E2 Proteins Reveals a New E2 Epitope with Significant Virus Neutralizing Activity
Source: PLoS Negl Trop Dis. 2010 Jul 13;4(7):e739. doi: 10.1371/journal.pntd.0000739 (PMC2903468; doi:10.1371/journal.pntd.0000739)
Supplement: Table S1 — Primers used in reverse transcription PCR and sequencing reactions. (0.03 MB DOC) [file pntd.0000739.s006.doc]

Table S1. Primers used in reverse transcription PCR and sequencing reactions.

| Primer^a^ | Sequence |
| --- | --- |
| 8134 | 5’-CACCCATGAGAAACCCCAAGGCTATT |
| 8644 | 5’-CCATAGTCCAATAGCAATCGAGGCAGTAAA |
| 9149 | 5’-GCCCTGGTGGAATGCGAGTG |
| 9698 | 5’-ACCGTTTCCGTTGCAGCGTCT |
| 9941 | 5’-AGGTGCGTGTGCTGTGTCGT |
| 10067 | 5’-GGCTACGCACCACTCCCTATCA |
| 10577 | 5’-GGGGCAGGACAACCAGGAG |
| 11051 | 5’-TCGGCGACTATCCATTTCTC |
| c8717 | 5’-GCGAGGAAGTCTGAAGTCTAACATAAC |
| c9228 | 5’-TTTGTGCACTGGCTGAACTGTTTTGTCTTG |
| c9300 | 5’-GCTTTGGGCAGTTTGTCAGAAT |
| c9813 | 5’-CAGCAAAGCACAGCCAGACA |
| c10380 | 5’-GCTGTGTGCGCTTTATATGCTTCA |
| c10933 | 5’-GTCGGAAGAATACACGCACTCG |
| c11421 | 5’-CGGAAAAGAAAAGAAAAATAAAATAAAAAT |

^a^Number indicates the position of the 5’ nucleotide of the primer based on the full-length Venezuelan equine encephalitis virus TC-83 sequence (Kinney, R. M., Johnson, B. J. B., Welch, J. B., Tsuchiya, K. R., and Trent, D. W., 1989. The full-length nucleotide sequences of the virulent Trinidad donkey strain of Venezuelan equine encephalitis virus and its attenuated vaccine derivative, strain TC-83. Virology 170, 19-30); “c” indicates a negative sense primer.
