# Supplementary material for: The First Human Epitope Map of the Alphaviral E1 and E2 Proteins Reveals a New E2 Epitope with Significant Virus Neutralizing Activity
Source: PLoS Negl Trop Dis. 2010 Jul 13;4(7):e739. doi: 10.1371/journal.pntd.0000739 (PMC2903468; doi:10.1371/journal.pntd.0000739)
Supplement: Table S2 — ELISA binding of purified human and murine Venezuelan equine encephalitis virus (VEEV) MAbs to either native or 0.3% β-propiolactone (BPL)-treated VEEV TC-83. (0.03 MB DOC) [file pntd.0000739.s007.doc]

Table S2. ELISA binding of purified human and murine Venezuelan equine encephalitis virus (VEEV) MAbs to either native or 0.3 % β-propiolactone (BPL)-treated VEEV TC-83.

| MAb | TC-83 virus | A_405_ at 10 µg MAb/ml^a^ | MAb endpoint concentration (ng/ml) |
| --- | --- | --- | --- |
| F5 nIgG^b^ | Native | 4.082 | 5 |
| F5 nIgG | BPL | 1.830 | 10 |
| F5 eIgG | Native | 4.066 | 5 |
| F5 eIgG | BPL | 1.026 | 20 |
| Hy4 IgG^c^ | Native | 4.084 | 2.4 |
| Hy4 IgG | BPL | 4.128 | 2.4 |
| 3B4C-4 | Native | 1.973 | 20 |
| 3B4C-4 | BPL | 1.746 | 10 |

^a^MAb starting concentration was 10 µg/ml.

^b^Human MAb F5 IgG was tested as both native (n) and engineered (e) antibody.

^c^Hy4 IgG is a humanized antibody derived from murine MAb 3B4C-4 (Hunt, A. R*.*, Frederickson, S., Hinkel, C., Bowdish, K. S., and Roehrig, J. T., 2006. A humanized monoclonal antibody protects mice either before or after challenge with virulent Venezuelan equine encephalomyelitis virus. J. Gen. Virol, 87, 2467-2476).
